# Genome-based polymorphic microsatellite development and validation in the mosquito *Aedes aegypti *and application to population genetics in Haiti

**DOI:** 10.1186/1471-2164-10-590

**Published:** 2009-12-09

**Authors:** Diane D Lovin, Katie O Washington, Becky deBruyn, Ryan R Hemme, Akio Mori, Sarah R Epstein, Brent W Harker, Thomas G Streit, David W Severson

**Affiliations:** 1 Eck Institute for Global Health, Department of Biological Sciences, University of Notre Dame, Notre Dame, IN 46556-5645 USA

## Abstract

**Background:**

Microsatellite markers have proven useful in genetic studies in many organisms, yet microsatellite-based studies of the dengue and yellow fever vector mosquito *Aedes aegypti *have been limited by the number of assayable and polymorphic loci available, despite multiple independent efforts to identify them. Here we present strategies for efficient identification and development of useful microsatellites with broad coverage across the *Aedes aegypti *genome, development of multiplex-ready PCR groups of microsatellite loci, and validation of their utility for population analysis with field collections from Haiti.

**Results:**

From 79 putative microsatellite loci representing 31 motifs identified in 42 whole genome sequence supercontig assemblies in the *Aedes aegypti *genome, 33 microsatellites providing genome-wide coverage amplified as single copy sequences in four lab strains, with a range of 2-6 alleles per locus. The tri-nucleotide motifs represented the majority (51%) of the polymorphic single copy loci, and none of these was located within a putative open reading frame. Seven groups of 4-5 microsatellite loci each were developed for multiplex-ready PCR. Four multiplex-ready groups were used to investigate population genetics of *Aedes aegypti *populations sampled in Haiti. Of the 23 loci represented in these groups, 20 were polymorphic with a range of 3-24 alleles per locus (mean = 8.75). Allelic polymorphic information content varied from 0.171 to 0.867 (mean = 0.545). Most loci met Hardy-Weinberg expectations across populations and pairwise F_ST _comparisons identified significant genetic differentiation between some populations. No evidence for genetic isolation by distance was observed.

**Conclusion:**

Despite limited success in previous reports, we demonstrate that the *Aedes aegypti *genome is well-populated with single copy, polymorphic microsatellite loci that can be uncovered using the strategy developed here for rapid and efficient screening of genome supercontig assemblies. These loci are suitable for genetic and population studies using multiplex-PCR.

## Background

The mosquito, *Aedes aegypti*, is the principal global vector for the yellow fever and dengue viruses, and also one of the best genetically characterized insects [1]. Of African origin, *Ae. aegypti *has successfully colonized most sub-tropical and tropical regions of the world, largely as a consequence of human activities. This mosquito has been and remains the most commonly studied mosquito species, particularly for genetic analyses of disease vector/pathogen interactions because it breeds in small water-holding containers, its eggs are resistant to desiccation and persist in a pre-embryonated state, and it readily adapts to laboratory culture. Detailed genetic studies have emerged from linkage maps for *Ae. aegypti *generated from isozyme and mutant marker loci [2], RAPDs [3], RFLPs [4,5], and SSCPs [6]. Demonstration that RFLP markers based on cDNAs had inter-specific utility [7] facilitated development of comparative linkage maps for several mosquito species [8-12].

Microsatellites are simple sequence repeats of tandem 1-6 base motifs that are frequently distributed throughout eukaryote genomes. Because repeat number at individual loci can vary among individuals and polymorphisms can efficiently be uncovered using PCR, microsatellites have become powerful tools for genetic studies in many organisms [13-15]. Of interest, useful microsatellite loci in some organisms including *Ae. aegypti *are not abundant or are recalcitrant to common methods of identification. In *Ae. aegypti*, these include microsatellite enriched genomic library construction and screening [16-18], examinations of expressed gene coding sequences [19,20], and oligonucleotide-based screening of select cosmid genomic clones [18]. Disappointingly, the combined efforts of these studies resulted in only 20 useful microsatellite marker loci, several of which showed reduced polymorphism. These results were most likely due to their close association with repetitive elements as opposed to microsatellite frequency in the *Ae. aegypti *genome [18]. Availability of a partial *Ae. aegypti *genome sequence in 2005 provided the opportunity to perform genome scans for microsatellites and, indeed, an additional 13 polymorphic microsatellites were uncovered [21].

Here we present a systematic approach to efficient polymorphic microsatellite marker development in *Ae. aegypti *based on intensive scans of supercontig assemblies from the whole genome shotgun sequence (wgs) assembly for *Ae. aegypti *[22]. In addition, we identified multiplex combinations of microsatellite loci that facilitate rapid genome-wide genotyping and demonstrate the utility of these microsatellite loci in a preliminary investigation of *Ae. aegypti *population genetic structure in Haiti.

## Results and Discussion

### Microsatellite identification, assays and utility

**Figure 1:**
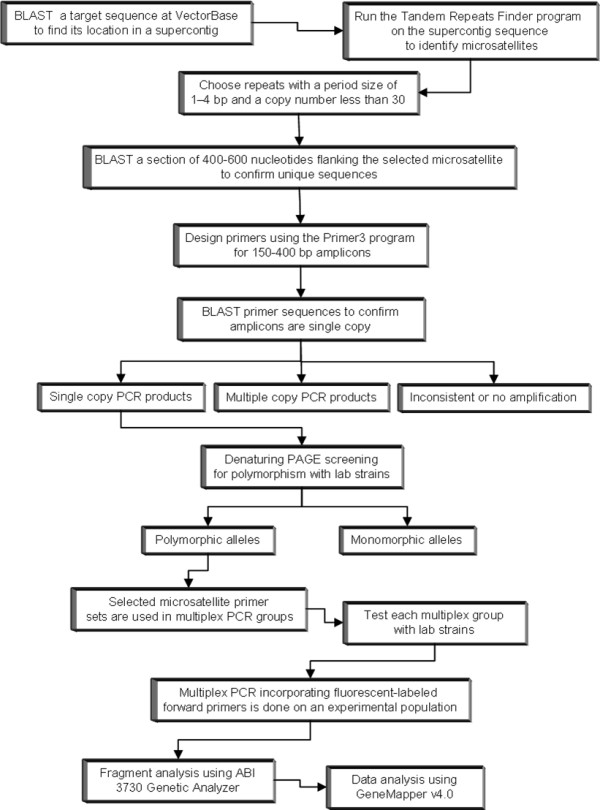
**Approach to genome-based microsatellite identification, validation, and analysis in *Aedes aegypti***.

Tandem Repeats Finder (TRF) [23] was used to systematically screen 42 wgs supercontig sequence assemblies in the *Ae. aegypti *genome for polymorphic single copy microsatellites (Figure 1). The supercontigs were selected on the basis of containing previously characterized genetic marker loci distributed across all three *Ae. aegypti *chromosomes [5]. Of 75 putative microsatellite loci tested, we determined that 44 amplified as single copy sequences in all or some of the four mosquito lab strains tested, of which 33 were found to be polymorphic across the four strains with a range of 2-6 alleles per locus (Additional File 1). These included 18 loci on chromosome 1, 5 loci on chromosome 2, and 10 loci on chromosome 3. Of the remaining 31 putative loci, 28 were determined to represent multicopy sequences and four sequences failed to amplify. In addition, four supercontigs contained no useful microsatellites based on our selection criteria. Chromosome locations for supercontigs and associated microsatellites were assigned based on the linkage map positions of the previously defined genetic loci. An additional 28 putative microsatellite loci amplified as multiple copies. No microsatellite sequences were evident in four supercontigs. Thus, direct scans of *Ae. aegypti *supercontigs provided a rapid and efficient mechanism for developing useful microsatellite loci and also the opportunity to leverage existing information on supercontig genome positions relative to the existing genetic linkage map. When coupled with the previously described 33 microsatellite loci [16,18-21], this effort has doubled the number of available polymorphic loci.

We tested microsatellites representing 31 motifs (1-6 bp); these included one single nucleotide (n = 3 sequences), five di-nucleotide (n = 27), 18 tri-nucleotide (n = 35), six tetra-nucleotide (n = 9), and one hexa-nucleotide (n = 1) motifs (Table 1). The single-copy polymorphic microsatellites comprise 22 independent motifs, of which 13 were tri-nucleotide motifs and these represented the majority (18 of 33) of the polymorphic single copy loci. Of particular note, 51% (18 of 35) of the tri-nucleotide and 67% (6 of 9) of the tetra-nucleotide microsatellites were polymorphic single copy loci, while only 33% (9 of 27) of the di-nucleotide microsatellites were polymorphic single copy loci. Although a small number of polymorphic tri-nucleotide microsatellite loci contained within coding regions have been identified in previous studies [20], BLAST analyses against the annotated *Ae. aegypti *genome assembly at VectorBase [24] indicated that none of our polymorphic tri-nucleotide microsatellites were within putative coding regions.

**Table 1:** Microsatellite loci PCR screen results categorized by repeat motif.

Repeat	Polymorphic (n = 33)	Monomorphic (n = 3)	Strain-specific amplification (n = 8)	Multiple copies (n = 28)	No amplification (n = 3)
**Single nucleotide repeats**					
A/T			2	1	
					
**Dinucleotide repeats**					
AG/TC	1			2	
AT/TA	2	1	1	9	1
CA/GT	1		1	1	1
CT/GA	4			1	
TG/AC	1				
					
**Trinucleotide repeats**					
AAC/TTG	1				
AAG/TTC	1			2	
AAT/TTA	2				
ACG/TGC	2				
ATA/TAT	1				
ATC/TAG				2	1
ATG/TAC	1				
ATT/TAA	1		1		
CAA/GTT			1		
CAG/GTC				1	
CAT/GTA	1			1	
CCA/GGT		1			
CGA/GCT	1		1	1	
CGT/GCA	2				
CTA/GAT		1		2	
CTT/GAA	3			1	
GAC/CTG	1			1	
TGA/ACT	1				
					
**Four or more nucleotide repeats**					
AATA/TTAT			1	1	
ATCC/TAGG	1				
ATGG/TACC	1				
ATTT/TAAA	3				
TGTA/ACAT	1				
TTTA/AAAT				1	
TGGACT/ACCTGA				1	

To improve the utility and efficiency of microsatellites for genotyping applications in *Ae. aegypti*, we developed seven groups of 4-5 loci each for multiplex-ready PCR [25] (Table 2). Individual loci in each group were selected to provide broad genome representation and relatively uniform amplification under the same PCR conditions when multiplexed. PCR groups 1A and 4A represent slight variants on groups 1 and 4, respectively: most of the loci are common among the respective groups with some inter-change of microsatellite loci that provide for potential diversity of chromosome coverage but still amplify well as multiplex PCR groups. Primers were designed to generate amplicons from ~150-400 bp and were fluorescently labeled for analysis by capillary electrophoresis. We included four microsatellite loci described elsewhere [18,21] in some of the groups. However, in conjunction with optimizing amplicon sizes for multiplex-ready PCR, we designed at least one new primer for each of these loci (Additional File 2).

**Table 2:** Multiplex-ready PCR groups.

Group	Map location^a^	Microsatellite Locus	Amplicon size (bp)	Fluorochrome
**1**	2-70.2	1132CT1	171	6-FAM^®^
	3-00.0	301CT1	267	NED^®^
	1-65.5	440TGTA1	294	6-FAM
	2-29.4	462GA1^b^	343	HEX^®^
				
**1A**	2-70.2	1132CT1	171	6-FAM
	3-50.0	217CTT1	257	NED
	2-29.4	462GA1^b^	343	HEX
	1-56.4	68GAC1	386	6-FAM
				
**1B**	2-70.2	1132CT1	171	6-FAM
	1-29.7	71AT1	191	NED
	3-64.2	470CT2	315	6-FAM
	2-29.4	462GA1^b^	343	HEX
				
**2**	2-07.3	328CTT1	229	6-FAM
	3-64.2	470AG1	252	HEX
	3-23.5	766ATT1	301	NED
	2-36.7	109CT1^c^	355	6-FAM
	1-29.7	71CGT1	387	HEX
				
**3A**	1-10.2	176TG1	166	HEX
	3-32.1	69TGA1	214	NED
	1-19.6	12ATG1	231	6-FAM
	3-14.6	288CTA1	321	6-FAM
	2-00.0	145TAAA1	335	HEX
				
**4**	1-19.6	12ACG1	177	6-FAM
	2-29.4	88AT1^c^	221	HEX
	3-43.7	86AC1^c^	257	NED
	3-00.0	301ACG1	287	6-FAM
	3-57.1	201AAT1	336	HEX
				
**4A**	1-19.6	12ACG1	177	6-FAM
	2-47.9	25AAG1	214	HEX
	3-43.7	86AC1^c^	257	NED
	3-00.0	301ACG1	287	6-FAM
	3-57.1	201AAT1	336	HEX

^a^Genetic map position after Severson et al. [5]

^b^Adapted from Chambers et al. [18] (Additional File 2)

^c^Adapted from Slotman et al. [21] (Additional File 2)

### Genetic patterns of *Aedes aegypti *populations in Haiti

We used multiplex-ready PCR groups 1, 1B, 2, 3A and 4 to conduct investigations of seven *Ae. aegypti *populations sampled in Haiti during June 2008. PCR groups 1A and 4A as variants on groups 1 and 4, respectively, were not included in our assays of samples from Haiti. Of the 23 microsatellite loci represented in these groups, 20 were polymorphic with a range of 3 to 24 alleles per locus with a mean of 8.75 alleles per locus among a total of 277 individuals across the seven sample sites (Table 3). Further, for 17 of the 20 polymorphic loci, at least 5 alleles were segregating among the populations. Allelic polymorphic information content (PIC) varied from 0.171 to 0.867 across loci with a mean PIC = 0.545 (Additional File 3), indicating that a large proportion of these loci are highly informative for population studies. Thus, these multiplex-ready PCR groups represent an efficient option for rapidly screening individual mosquitoes in 96-well microplate format. Our genotype analyses of mosquito collections from Haiti with the selected multiplex-ready PCR groups indicated that these microsatellite loci exhibited higher levels of polymorphism compared with previous microsatellite data reported for *Ae. aegypti *field populations in Côte d'Ivoire, Kenya and Vietnam, respectively [19,21,26].

**Table 3:** Unique alleles in a Haiti population (n = 277 progeny).

Multiplex group	SSR Locus	# of alleles	Size range (bp)
**1**	1132CT1	24	151-203
	301CT1	11	262-282
	462GA1	13	331-359
			
**1B**	71AT1	3	186-190
	470CT2	6	312-322
			
**2**	328CTT1	7	213-233
	470AG1	8	227-255
	766ATT1	5	300-320
	109CT1	14	332-358
	71CGT1	5	380-392
			
**3A**	176TG1	15	136-172
	69TGA1	4	212-218
	12ATG1	7	205-233
	288CTA1	3	311-321
	145TAAA1	11	318-350
			
**4**	12ACG1	6	167-179
	88AT1	14	219-248
	86AC1	5	253-261
	301ACG1	5	279-287
	201ATT1	8	324-346

The observed heterozygosity was generally high among all loci and across the seven populations, with notable exceptions at three loci (Additional File 3). The 301CT1, 328CTT1, and 145TAAA1 loci each showed very low, and in several populations, no heterozygosity. While we have no explanation for this phenomenon, it is interesting to note that each of these loci is located at or near the end of a linkage group; the associated supercontigs contain the genetic loci LF347, LF115, and AEGI8, respectively [5]. However, after excluding these three loci, most loci met Hardy-Weinberg (HW) expectations, with the exception of the Port au Prince population where seven of the remaining 17 loci showed significant HW deviations. The observed HW deviations across all populations were due to heterozygote deficits.

Significant population differentiation was observed with 10 of 21 (48%) pairwise F_ST _comparisons (Table 4). The mean pairwise F_ST _across the 20 polymorphic loci ranged from 0.014 (La Poudriere and Grand Goave) to 0.104 (Grand Goave and Barriere-Jeudy). Port au Prince showed significant differentiation with four of the other six populations, while the La Poudriere population showed no differentiation with any of the other populations. To test for isolation by distance, we regressed the pairwise F_ST_/(1-F_ST_) against the natural logarithm of the distance between sites (Additional File 4). We found no association between them (R^2 ^= 0.0355, P = 0.41). That is, while distances between sites varied from ~1.4 to 44.5 km, the observed levels of genetic differentiation between sites were sometimes high and sometimes low irrespective of distance. This result is typical for *Ae. aegypti *populations as adults generally travel very short distances from breeding sites in a lifetime, often ~100 m or less, with some evidence for greater but still modest dispersal (~800 m) [27-31]. Longer range dispersal and population differentiation are more likely to reflect the effects of mosquito transport via human activities than relative distances among breeding sites and active dispersal by individual mosquitoes.

**Table 4:** Pairwise F_ST _estimates for populations from Haiti based on 20 microsatellite loci

	La Poudriere	Grand Goave	Bino	Ca-Ira	Barriere-Jeudy	Chawa
Port au Prince	0.051	0.042*	0.025	0.059*	0.046*	0.026*
La Poudriere		0.014	0.039	0.092	0.097	0.086
Grand Goave			0.062	0.080*	0.104*	0.077*
Bino				0.063	0.033	0.033
Ca-Ira					0.074*	0.066*
Barriere-Jeudy						0.047*

*P < 0.05 using Bonferroni adjusted significant thresholds to account for multiple testing.

## Conclusion

We demonstrate that the *Ae. aegypti *genome is well-populated with microsatellite loci suitable for genotyping and outline an efficient strategy for identifying and validating microsatellites from genome supercontig assemblies. While multiple repeat motifs were evident and represented as single copy sequences, tri-nucleotide microsatellites were the most common, and with tetra-nucleotide microsatellites, the most applicable to development as genetic loci. We developed several multiplex-ready PCR groups of microsatellite loci that permit rapid genotyping, and demonstrate their utility with *Ae. aegypti *population samples from Haiti. We observed high polymorphism with a mean of 8.75 alleles per locus, high allelic polymorphic information content (PIC), and evidence for population differentiation even across relatively short geographic distances as is often reported for *Ae. aegypti*.

## Methods

### Mosquito strains and populations

Preliminary screens of microsatellites for single copy number and polymorphism were evaluated among individuals from four *Ae. aegypti *laboratory colonies, Liverpool-IB12, MOYO-R, Trinidad, and Haiti. The laboratory strains have been maintained as colonies for an unknown number of generations and likely carry reduced polymorphism compared to field-collected individuals. The Liverpool-IB12 strain was the source for the *Ae. aegypti *genome project [22], details on the MOYO-R and Trinidad strains are provided elsewhere [32], and the Haiti strain was established from ovitrap samples collected in 2006.

Field samples from Haiti were collected from three localities (Port-au-Prince, Grand Goave, and Leogane) during June, 2008; samples from Leogane were collected at five different regions in the city (Barriere-Jeudy, Bino, Ca-Ira, Chawa, La Poudriere). Port au Prince and Grand Goave were separated by the greatest distance (~44.5 km). All sites in Leogane were within ~10 km of each other with La Poudriere and Ca-Ira being the closest (~1.3 km). At each site, samples included larval collections from containers around households, standard ovitrap collections with 10 traps at each site, or both larval and ovitrap collections. Ovitrap sampling and mosquito rearing were performed generally as reported previously [33]. Genotype data for all individuals obtained at each sample site were pooled for subsequent analysis.

### *In silico *identification of microsatellites in the *Aedes aegypti *genome assembly

Bioinformatic analyses targeted the identification and development of useful microsatellite loci at ~10 cM intervals across each of the three *Ae. aegypti *chromosomes. Supercontig assemblies for microsatellite scans were identified by BLASTn analysis against the *Ae. aegypti *genome (version AaegL1, March 2006) at VectorBase [24] with sequences previously mapped as RFLP, SNP and SSCP genetic markers [5]. Supercontig assemblies containing individual marker loci were then downloaded from VectorBase and screened with the Tandem Repeats Finder (TRF) program using default parameters [23]. The TRF output was manually scanned and, in most cases, tandem repeats with a period size of 2-4 bp and repeat copy number less than 30 were arbitrarily selected for further analysis.

### Primer Design

In preparation for primer design, a ~400-600 bp sequence containing a microsatellite of interest was extracted from the supercontig sequence and subjected to BLASTn analysis against the *Ae. aegypti *genome sequence at VectorBase to verify that the microsatellite flanking sequences were not highly repetitive. PCR primers were designed for those sequences showing minimal repetitive sequence using Primer3 v.4.0 [34], with the amplicon size target set at 150-400 bp. Individual primer pairs selected from the Primer3 output were also subjected to BLAST analysis to verify that they represented single copy sequences in the *Ae. aegypti *genome.

### PCR Amplification

DNA extractions on individual mosquitoes were performed following a rapid, simple alkaline method [35]. DNA was suspended in a final volume of 1600 μl containing 0.01 M NaOH and 0.018 M Tris-HCl, pH 8.0. Amplification was performed in 25 μl volumes in 96-well PCR plates (Dot Scientific) in a Mastercycler thermocycler (Eppendorf). Each reaction contained 1× Taq buffer (50 mM KCl, 10 mM Tris pH 9.0, 0.1% Triton X), 1.5 mM MgCl_2_, 200 μM dNTPs, 5 pmoles of each primer, 1 unit of Taq DNA polymerase, and 1 μl of genomic DNA as prepared above. Thermocycling conditions were: 5 minutes at 94°C, followed by 30 cycles of a 1 minute denaturation at 94°C, a 1 minute anneal at 60°C, a 2 minute extension at 72°C, followed by a 10 minute final extension cycle at 72°C. PCR products were size fractionated by electrophoresis in 2% agarose gels stained with ethidium bromide, and visualized under UV light.

### Polymorphism Determination and Multiplex PCR

Microsatellites with single copy amplicons based on agarose gel screens were assayed for allelic polymorphisms on 6% denaturing polyacrylamide gels using the *GenePrint*^® ^STR System (Promega). Data for single copy sequences have been submitted to the GenBank STS database (Additional File 5). Select primer pairs for loci that showed polymorphism among strains were evaluated and assembled into multiplex groups of four or five loci per group. Multiplex group criteria included efforts to combine microsatellite loci that provided broad coverage across each chromosome and exhibited detectable amplicon size differences on agarose gels. Multiplex groups were tested for amplification with DNA from single mosquitoes in 25 μl PCR reactions as outlined above.

### Fragment Analysis and Genotyping

Flurochrome-labeled forward primers (6-FAM^®^, HEX^®^, NED^®^) were synthesized by Integrated DNA Technologies and Applied Biosystems for each primer pair that successfully amplified in the multiplex group. Multiplex PCR products were diluted 1:10 in sterile water and 1 μl of this dilution was added to 9 μl of a mixture of HiDi Formamide^® ^(ABI #4311320) and ROX 400HD^® ^standard (ABI #402985) in 96 well PCR plates. The samples were then denatured for 2 minutes at 95° and immediately placed on ice. Plates were kept covered during processing due to the light-sensitive standard and dye-labeled primers. Genotyping was performed using an ABI 3730 Genetic Analyzer with the GeneMapper^® ^v.4.0 software package.

### Population data analysis

Analysis of genetic diversity among *Ae. aegypti *populations from Haiti included calculations of the observed and expected heterozygosities and number of alleles per locus. F_IS _(inbreeding coefficient) and F_ST _(fixation index) were estimated following Weir and Cockerham [36] using FSTAT 2.9.3 [37,38]. Deviations from Hardy-Weinberg expectations were determined using FSTAT 2.9.3. Critical significance levels were adjusted for multiple comparisons using Bonferroni corrections. Allelic polymorphic information content (PIC) was calculated using the Excel Microsatellite Toolkit 3.3.1 [39]. PIC = 1 - Σ(P_ij_)^2 ^where P_ij _is the frequency of the *i*th allele in the *j*th population for each locus. Genetic isolation by distance was evaluated by linear regression of the pairwise F_ST _values as F_ST_/(1-F_ST_) on the natural logarithm transformation of the distance between sites using R version 2.6.0 [40].

## Abbreviations

TRF: Tandem Repeats Finder; wgs: whole genome sequence; PIC: polymorphic information content; HW: Hardy-Weinberg.

## Authors' contributions

DDL coordinated the project and drafted the manuscript. KOW carried out laboratory assays and data analysis for the Haiti populations. BD assisted with microsatellite characterization and developed the multiplex-ready PCR assays. RRH assisted with microsatellite identification and characterization and data analysis. AM and SRE assisted with microsatellite identification and characterization. BWH assisted with microsatellite analysis. TGS coordinated collection of samples from Haiti. DWS planned and supervised the project and assisted in writing the manuscript. All authors read and approved the final manuscript.

## Supplementary Material

Additional file 1 **Microsatellite variation in lab strains**. The data provided include detailed information on all microsatellite sequences screened for copy number and polymorphism against four laboratory strains, and include primer sequences for each locus. Click here for file

Additional file 2 **Re-designed PCR primer sequences**. The data provided represent the complete descriptions for the previously reported microsatellite loci for which new PCR primers were designed. Click here for file

Additional file 3 **Microsatellite polymorphisms among field populations from Haiti**. The data provided represent the locus by population statistics that include allele numbers, heterozygocities, Hardy-Weinberg expectations, and allelic polymorphic information content. Click here for file

Additional file 4 **Regression analysis of pairwise F_ST_/(1-F_ST_) against pairwise natural logarithm-transformed distances among sample sites in Haiti**. The data provided represent the comparison of microsatellite-based population structure to distances between populations in Haiti. Click here for file

Additional file 5 **GenBank accession numbers for STS sequences of microsatellite loci**. The data provided represent the complete list of single copy microsatellite loci with the associated GenBank Sequence Tagged Site (STS) database accession numbers. Click here for file
