# Supplementary material for: Genome-based polymorphic microsatellite development and validation in the mosquito Aedes aegypti and application to population genetics in Haiti
Source: BMC Genomics. 2009 Dec 9;10:590. doi: 10.1186/1471-2164-10-590 (PMC3087561; doi:10.1186/1471-2164-10-590)
Supplement: Additional file 1 — Microsatellite variation in lab strains. The data provided include detailed information on all microsatellite sequences screened for copy number and polymorphism against four laboratory strains, and include primer sequences for each locus. [file 1471-2164-10-590-S1.DOC]

| **Additional File 1: Microsatellite variation in lab strains.** | | | | | |  | |  | |  | |  | |  | |
| --- | --- | --- | --- | --- | --- | --- | --- | --- | --- | --- | --- | --- | --- | --- | --- |
|  | |  | |  |  | |  | |  | |  | |  | |  |
| **Supercontig #** | | **Microsatellite locus^a^** | | **Genetic locus^b^** | **GenBank accession #** | | **Map location^c^** | | **Predicted amplicon size (bp)** | | **Forward primer 5'-3'** | | **Reverse primer 5'-3"** | | **Allele #** |
|  | |  | |  |  | |  | |  | |  | |  | |  |
| **Single copy polymorphic (n=33)** | | | |  |  | |  | |  | |  | |  | |  |
| 1.176 | | 176CAT1 [8] | | Tfs | AF019117 | | 1-10.2 | | 214 | | CCCTTTTGTCCCATCTCTCA | | ACTGGTCTACCGATGGCAAC | | 2 |
| 1.176 | | 176TG1 [18] | | Tfs | AF019117 | | 1-10.2 | | 166 | | CGATCGTTGAAGGCATTTTT | | GAGGAACGAAACGCCAATTA | | 6 |
| 1.12 | | 12ACG1 [7] | | AEGI30 | BI096856 | | 1-19.6 | | 177 | | GTCACCCCAAGTAGCTTTGC | | GAGGCTTTACTGCTGGTTCG | | 3 |
| 1.12 | | 12ATG1 [11] | | AEGI30 | BI096856 | | 1-19.6 | | 231 | | TCTGCGCAACACTAAGCATC | | AAAAAGCACACCGTTCTCGT | | 3 |
| 1.71 | | 71CGT1 [8] | | AeW | U73826 | | 1-29.7 | | 387 | | CACCCATTACTTGCCGTTCT | | CTCCCCCAAACTCTTTAGCC | | 2 |
| 1.71 | | 71AT1 [11] | | AeW | U73826 | | 1-29.7 | | 191 | | GCACTGCTCAAAATCACCAC | | CAACACGCTCCATCCAGTTA | | 3 |
| 1.344 | | 344ATTT1 [14] | | B8L260 | BH817084 | | 1-30.0 | | 338 | | GAATTTTTCAGAATGACGATTGA | | GTGTGAAGGGATTTGGCATT | | 2 |
| 1.192 | | 192TAAA1 [7] | | LAP | M95187 | | 1-36.6 | | 273 | | TTCTACTCCAGGATTCAAACAAG | | TGTAGCGGCCATGAGTGTTC | | 2 |
| 1.335 | | 335CGA1[11] | | ARC3 | BH214542 | | 1-38.2 | | 479 | | GTTCTCGTCCCAAAGTGCAT | | AAGCTAGGATGGTGGCAATG | | 2 |
| 1.12 | | 12CGT1 [8] | | LF150 | BM005476 | | 1-40.7 | | 307 | | CCTGCTGCCTTATTTCGTTG | | CACTGCTAGCGGATTGCATA | | 3 |
| 1.88 | | 88CA1 [16] | | TY7 | R19560 | | 1-44.9 | | 435 | | CACGCTGCGTATTTGATGTT | | GGATGCTGCTTTCGTTTCTC | | 3 |
| 1.88 | | 88GAA1 [8] | | TY7 | R19560 | | 1-44.9 | | 252 | | TGGCATGTTTGATTGACTCC | | AACGACATGGCGACCTACTT | | 3 |
| 1.673 | | 673TA1 [17] | | CHT2 | AF026492 | | 1-49.9 | | 311 | | GATGAAAGCTCATCAATGTTTCAG | | TGGGCTCCAAATTTCAGAAG | | 3 |
| 1.68 | | ATGG1 [6] | | AEGBS11 | AY033622 | | 1-56.4 | | 345 | | ATGTGCCCTATCGACGATTC | | TCTTCTTCTTCGGCTTTGGA | | 2 |
| 1.68 | | 68GAC1 [8] | | AEGBS11 | AY033622 | | 1-56.4 | | 386 | | CGTGTCCGGTGGACTATTCT | | AACTTTTCGGTGAACCATCG | | 4 |
| 1.440 | | 440AAC1 [12] | | RT6 | BH214544 | | 1-65.5 | | 358 | | CCCCAATCATCAGATTACCG | | ACATCGAGTGTGCAGAGTGC | | 2 |
| 1.440 | | 440ATCC1 [8] | | RT6 | BH214544 | | 1-65.5 | | 272 | | CTTGGTTTTCCCTTCGATCA | | ACCCTCCGTTCAAGTTGATG | | 2 |
| 1.440 | | 440TGTA1 [12] | | RT6 | BH214544 | | 1-65.5 | | 294 | | GGTCGTCTGGGGTGAAATAA | | GCGCATTTAAACGTGACAAG | | 3 |
| 1.145 | | 145TAAA1 [11] | | AEGI8 | AF326340 | | 2-00.0 | | 335 | | AGCCTTGGTATGGGGAATTT | | AGCATTCTTAGAGCGCTGGT | | 4 |
| 1.25 | | 25AAG1 [15] | | LF138 | T58332 | | 2-47.9 | | 214 | | CTTGCCACACGATGCTAAAA | | TGAATTCAAACCCATACGTGA | | 3 |
| 1.121 | | 121GA1 [11] | | Ef2 | AY040342 | | 2-59.8 | | 175 | | CTACCAGCCTTGCGTTTTTG | | GGTTCTCCGCAGATATTTTTC | | 4 |
| 1.328 | | 328CTT1 [7] | | LF115 | R67978 | | 2-07.3 | | 229 | | ATCCGCTGGATTTACAATGG | | CTGTCCAAGTGCGAACGTAA | | 4 |
| 1.1132 | | 1132CT1 [14] | | BA67 | AI561370 | | 2-70.2 | | 171 | | TGACGCGTTTGACGTAGTTT | | TCCGCAATTACGCTAACAAA | | 5 |
| 1.301 | | 301ACG1 [7] | | LF347 | T58329 | | 3-00.0 | | 287 | | TTTCCGCGATGGAGAAATAC | | CAACCGGAAGACAGAAAAGG | | 3 |
| 1.301 | | 301CT1 [13] | | LF347 | T58329 | | 3-00.0 | | 267 | | CTGAACGCGCCATAAATTCT | | AGGAGTTCGTCCCAAGACAA | | 3 |
| 1.17 | | 17ATA1 [8] | | LF232 | BM005489 | | 3-22.4 | | 207 | | CGGAGATGAACAGCCATAGG | | CACCAGGAATCCACAAAAGG | | 3 |
| 1.766 | | 766ATT1 [7] | | LF103 | BM005488 | | 3-23.5 | | 301 | | TGCAAAGTCGAAGCAACAAG | | GAATGCCATTTGCCTTCA | | 3 |
| 1.69 | | 69TGA1 [8] | | LF168 | R47184 | | 3-32.1 | | 214 | | CACCTCCGCTAGAGAACTGG | | CGAATAGGGCAATCCTGAAA | | 2 |
| 1.217 | | 217CTT1 [10] | | Rpl31 | AF324863 | | 3-50.0 | | 257 | | TGGACTTCCCCAGATGCAATGA | | CAACACGGAAGCAAAGTTGA | | 2 |
| 1.201 | | 201TTA1 [8] | | Apy1 | L12389 | | 3-57.1 | | 214 | | AAACGATTGATCGTGTCGTG | | AGTCATGAATTGCCGGTCTT | | 2 |
| **Table 1: Microsatellite variation in lab strains.** *(Continued)* | | | | | | | | | | |  | |  | |  |
| 1.201 | | 201AAT1 [9] | | Apy1 | L12389 | | 3-57.1 | | 336 | | GATCGTTCGACAGCATCTGA | | GGAAAGCTCATCGCCTACTG | | 3 |
| 1.470 | | 470CT2 [9] | | PABP | AY038043 | | 3-64.2 | | 315 | | GTTGGGAGTGTCCGCATAGT | | GCTCCGGTCATCTTGAATGT | | 2 |
| 1.470 | | 470AG1 [12] | | PABP | AY038043 | | 3-64.2 | | 252 | | CGGGAGGACACACTGACTTT | | GTGGCAAGTAACCCCAAAGA | | 3 |
|  | |  | |  |  | |  | |  | |  | |  | |  |
| **Single copy monomorphic or strain-specific amplification (n=11)** | | | | | | | | |  | |  | |  | |  |
| 1.12 | | 12CAA1 [19] | | LF150 | BM005476 | | 1-40.7 | | 125 | | CTGTTGCTCCAGCAAGATGA | | CCCAACAGTCTTACACATCAAGA | | L,T^d^ |
| 1.446 | | 446CCA1 [10] | | LF284 | BM0055002 | | 1-38.0 | | 327 | | TATCCATCGTATTGCGGTTGA | | CGTCATCGTCGCTGCTGTCG | | N |
| 1.68 | | 68ATT1 [7] | | AEGBS11 | AY033622 | | 1-56.4 | | 276 | | TGTGCACCAAGAAAAATCGT | | ATCTTCCACGAACGAAATGC | | H, L |
| 1.438 | | 438A1 [14] | | AEGI22 | BI099650 | | 1-59.0 | | 101 | | ACCGACATTGTCAAACGAAA | | GTTCGTGAGATCCCCTTGAA | | H, L, M |
| 1.319 | | 319A1 [64] | | Rdl | U28803 | | 2-17.8 | | 292 | | CACGTAGGGTCGCTTCAGAT | | CAGATTATCCACCGGCATTC | | L |
| 1.25 | | 25TTAT1 [8] | | LF138 | T58332 | | 2-47.9 | | 195 | | AATCCAACTAACGCCCACTG | | CTGTGCCCCTTTCTTCAAAA | | H, L, M |
| 1.29 | | 29AT1 [15] | | CRALBP | AF329893 | | 2-54.4 | | 216 | | CCGTTTCATCAACGGTTAGG | | CGAATGCTGCGATCAACTTT | | N |
| 1.14 | | 14GCT1 [14] | | D6L600 | BH214535 | | 2-61.8 | | 349 | | GCGCTAGCGGTGTTACAACT | | CGATGCTCATAAGGGATCGT | | L |
| 1.288 | | 288CTA1 [23] | | AEGI28 | BI096849 | | 3-14.6 | | 321 | | AGGGAATGACGTCCTCACAG | | TTGGTTCTATTGGGCTTTGC | | N |
| 1.69 | | 69AT1 [39] | | LF168 | R47184 | | 3-32.1 | | 170 | | TCTCAATGGAAGTGCACACA | | ACAATAAGGAGTCGCCCTTG | | H, L, M |
| 1.69 | | 69CA1 [9] | | LF168 | R47184 | | 3-32.1 | | 229 | | GAGCTATCACCTTTCTGGGCTA | | GCTCGAAAATTACGCTCCTG | | L |
|  | |  | |  |  | |  | |  | |  | |  | |  |
| **Multiple copy PCR products (n=28)** | | | | |  | |  | |  | |  | |  | |  |
| 1.148 | 148AAG1 [9] | | | LF90 | T58320 | | 1-00.0 | | 272 | | ACGAATGCGAAAAGGACAAC | | TAAATCATGGGGACCAGCTC | |  |
| 1.148 | 148AG3 [8] | | | LF90 | T58320 | | 1-00.0 | | 275 | | AAAAGATGGCGACGAAAAGA | | ACCGCCATCATAGGATTCTG | |  |
| 1.148 | 148AT2 [27] | | | LF90 | T58320 | | 1-00.0 | | 319 | | TCATAGCATTGGAGCATGGA | | TCTGCAAGACGGAAATTCG | |  |
| 1.148 | 148AT1[24] | | | LF90 | T58320 | | 1-00.0 | | 282 | | GGGTCCGGAATGTACACAGA | | GAAATTCGCATACCAACTCG | |  |
| 1.209 | 209AT1 [63] | | | LF211 | BM005514 | | 1-11.5 | | 253 | | TTCACTTTGAGGCGAATGCT | | TTGGAGTTCTCTCGAAGCAAG | |  |
| 1.209 | 209CA2 [10] | | | LF211 | BM005514 | | 1-11.5 | | 221 | | ATGCATGGGAATTTTTGAGG | | CCCTATTCATCCACGTTTGC | |  |
| 1.209 | 209CAT3 [6] | | | LF211 | BM005514 | | 1-11.5 | | 225 | | ACTCACTGGCACACAACCAA | | AGCAGCGGGTTTTTCTTTTT | |  |
| 1.153 | 153TGGACT1 [16] | | | D6L500 | BH214541 | | 1-39.2 | | 398 | | TGACCTGTGGTAAATTGCTTG | | AGAACAAAGCCGAAGCTCAA | |  |
| 1.12 | 12TTTA1 [11] | | | LF150 | BM005476 | | 1-40.7 | | 132 | | ATTTTTGGGCATTTCACGAC | | TCTATGTCGAAGGCGGTGAT | |  |
| 1.1051 | 1051AATA1 [8] | | | nAcBP | AY040341 | | 1-44.5 | | 147 | | GGGCTACAAATTGCCTGTTG | | CCGTGTTGCAATTTTCACAT | |  |
| 1.465 | 465TA1 [26] | | | nAcBP | AY040341 | | 1-44.5 | | 120 | | CATGGAACCAAAACAACCATG | | TGTTTCTGTCGCGGTACCTA | |  |
| 1.739 | 739CGA1 [10] | | | APN | AF378117 | | 1-53.6 | | 184 | | AGAACGACACGACACACTCG | | ATTTCGGGTTCATTTTGTGC | |  |
| 1.145 | 145AG1 [8] | | | AEGI8 | AF326340 | | 2-00.0 | | 239 | | TTGTTCGTCAGCATCGAGAC | | GAACGCTCGGCATTGTTTAT | |  |
| 1.145 | 145AT1 [12] | | | AEGI8 | AF326340 | | 2-00.0 | | 380 | | CGAGCATTGGGTGTCAGTAA | | TTGGCTGAATTAGGGACGTT | |  |
| 1.328 | 328AT1 [63] | | | LF115 | R67978 | | 2-07.3 | | 218 | | TGGAAAAAGGGAAGGGATTT | | CAATTCCAGATTTCACCGATA | |  |
| 1.319 | 319CAG1 [8] | | | Rdl | U28803 | | 2-17.8 | | 157 | | GGTGGAAAACTCCCCGTATT | | GTACGCGATTGGTTGGTTTT | |  |
| 1.25 | 25GAT1 [12] | | | LF138 | T58332 | | 2-47.9 | | 163 | | AAAAAGTAAGGCGACGCAGA | | ACGCCTTGCAACCACTAAAC | |  |
| **Table 1: Microsatellite variation in lab strains.** *(Continued)* | | | | | | | | | | |  | |  | |  |
| 1.121 | 121GAT1 [8] | | | Ef2 | AY040342 | | 2-59.8 | | 213 | | TCCATCCGCAGTTCTCTTCT | | GGGGAGCAATGCTAAACAGA | |  |
| 1.14 | 14AT1 [24] | | | D6L600 | BH214535 | | 2-61.8 | | 292 | | TGGCTCAAAACGTTAATCACC | | ACGTTCGGCATAAAGCAGTT | |  |
| 1.14 | 14GAC1 [8] | | | D6L600 | BH214535 | | 2-61.8 | | 135 | | GCTCAGTCCCGAAACCATAA | | CACATTCACAACCCCAATGA | |  |
| 1.1132 | 1132TAG1 [9] | | | BA67 | AI561370 | | 2-70.2 | | 177 | | AAACGTTACGATTGCCGATT | | GTTTCTTGGGCTGGTAGACG | |  |
| 1.301 | 301AAG1 [32] | | | LF347 | T58329 | | 3-00.0 | | 354 | | GACGATTCAAAGCACACGAA | | AAGTAAGTGGGGCAAGCTGA | |  |
| 1.288 | 288ATC1 [13] | | | AEGI28 | BI096849 | | 3-14.6 | | 213 | | TACCACATTCGCCCACTTCT | | AGTAGGCTGGAGGGTTGGTT | |  |
| 1.766 | 766A1 [75] | | | LF103 | BM005488 | | 3-23.5 | | 399 | | AAAGAAATGGTCGGGCAAA | | ACACATTTTGGCGACAGTGA | |  |
| 1.766 | 766AT1 [26] | | | LF103 | BM005488 | | 3-23.5 | | 348 | | TGGTGATAACAAACGGACGA | | TAATTCTACGCAGGCAATTCAA | |  |
| 1.766 | 766CTT1 [40] | | | LF103 | BM005488 | | 3-23.5 | | 318 | | TCTCCTCAAATCGCCAAATC | | CGCTGAGTAACCGGATCTGT | |  |
| 1.201 | 201TA1 [32] | | | Apy1 | L12389 | | 3-57.1 | | 208 | | TGTCTGCCCAAAATTTCACA | | CGATCAGTTCGGTCTTCCAG | |  |
| 1.470 | 470CT1 [30] | | | PABP | AY038043 | | 3-64.2 | | 319 | | ATTTGTGGATGGTGCCAGAT | | CTCAATGGTCCTCGATGGTT | |  |
|  |  | | |  |  | |  | |  | |  | |  | |  |
| **No amplification or no usable microsatellites identified (n=7)** | | | | | | | | |  | |  | |  | |  |
| 1.710 | | | none | LF198 | T58319 | | 1-20.0 | |  | |  | |  | |  |
| 1.145 | | | 145TAG1 [8] | AEGI8 | AF326340 | | 2-0.0 | | 209 | | CTCTTCGTATCACGGACACC | | AGGTGTGAAGTTTGGAGCAGA | |  |
| 1.581 | | | none | LF250 | T58310 | | 2-16.5 | |  | |  | |  | |  |
| 1.861 | | | none | LF338 | BM005508 | | 2-18.6 | |  | |  | |  | |  |
| 1.507 | | | none | LF275 | BM005500 | | 2-44.0 | |  | |  | |  | |  |
| 1.14 | | | 14CA1 [22] | D6L600 | BH214535 | | 2-61.8 | | 164 | | ATCTCTAGTTGGGCGCGTTA | | GGTGGTCAATTGATGGGTTT | |  |
| 1.1232 | | | 1232AT1 [15] | BA67 | AI561370 | | 2-70.2 | | 245 | | TCGAAGGCATTTGTTTCCAC | | CGTGCAATGGTCAATATTCC | |  |

^a^[ ]: number of repeats; ^b^Genetic loci associated with supercontig; ^c^Genetic map position after Severson et al., 2002; ^d^Strain-specific amplification, H=Haiti, L=Liverpool-IB12, M=MOYO-R, T=Trinidad, N=amplification with all strains but monomorphic.

^c^Severson DW, Meece JK, Lovin DD, Saha G, Morlais I: **Linkage map organization of expressed sequence tags and sequence tagged sites in the mosquito, *Aedes aegypti*.** *Insect Mol Biol* 2002, **11**:371-378.
