# Supplementary material for: Genome-based polymorphic microsatellite development and validation in the mosquito Aedes aegypti and application to population genetics in Haiti
Source: BMC Genomics. 2009 Dec 9;10:590. doi: 10.1186/1471-2164-10-590 (PMC3087561; doi:10.1186/1471-2164-10-590)
Supplement: Additional file 2 — Re-designed PCR primer sequences. The data provided represent the complete descriptions for the previously reported microsatellite loci for which new PCR primers were designed. [file 1471-2164-10-590-S2.RTF]

Additional File 2.  Re-designed PCR primer sequences.


Supercontig #	Microsatellite locusa	GenBank accession #	Map locationb	Predicted amplicon size (bp)	Forward primer 5'-3'	Reverse primer 5'-3"	
							
1.462c	462GA1 [14]	DU169902	2-29.4	343	CAAACAACGAACTGCTCACG	GAATAAGTCACACGCGTCCA	
1.86d	86AC1 [5]	DV309356	3-43.7	257	GCGAATCGGTTCCCATAGTA	ACCCATCGAATTTCCATTCA	
1.88d	88AT1 [9]	DV389063	2-29.4	221	CGTCGACGTTATCTCCTTGTT	CCAACGCAAGATGCAAGATA	
1.109d	109CT1 [6]	DV362806	2-36.7	355	ACTGAACGCCAACCAAGC	AACACCATTCTTCAGCAGAT	
a[ ]: Number of repeats; bGenetic map position after Severson et al. [1]; cAdapted from Chambers et al. [2]; dAdapted from Slotman et al. [3].

1.	Severson DW, Meece JK, Lovin DD, Saha G, Morlais I: Linkage map organization of expressed sequence tags and sequence tagged sites in the mosquito, Aedes aegypti.  Insect Mol Biol 2002, 11:371-378.
2.	Chambers EW, Meece JK, McGowan JA, Lovin DD, Hemme RR, Chadee DD, McAbee K, Brown SE, Knuson KL, Severson DW: Microsatellite isolation and linkage group identification in the yellow fever mosquito Aedes aegypti.  J Hered 2007, 98:202-210.
3.	Slotman MA, Kelly NB, Harrington C, Kitthawee S, Jones W, Scott TW, Cacoone A, Powell JR: Polymorphic microsatellite markers for studies of Aedes aegypti (Diptera: Culicidae), the vector of dengue and yellow fever.  Mol Ecol Notes 2007, 7:168-171.
