# Supplementary material for: Genome-based polymorphic microsatellite development and validation in the mosquito Aedes aegypti and application to population genetics in Haiti
Source: BMC Genomics. 2009 Dec 9;10:590. doi: 10.1186/1471-2164-10-590 (PMC3087561; doi:10.1186/1471-2164-10-590)
Supplement: Additional file 3 — Microsatellite polymorphisms among field populations from Haiti. The data provided represent the locus by population statistics that include allele numbers, heterozygocities, Hardy-Weinberg expectations, and allelic polymorphic information content. [file 1471-2164-10-590-S3.DOC]

| **Additional File 3: Microsatellite polymorphisms among field populations from Haiti.** | | | | | | | | | |  |  |  |
| --- | --- | --- | --- | --- | --- | --- | --- | --- | --- | --- | --- | --- |
|  |  |  |  |  |  |  |  |  |  |  |  |  |
|  | **Port au Prince** | |  | **La Poudriere** | |  | **Grand Goave** | |  | **Bino** |  |  |
|  |  |  |  |  |  |  |  |  |  |  |  |  |
| Locus | n^a^ | H_obs_^b^ | F_IS_^c^ | n | H_obs_ | F_IS_ | n | H_obs_ | F_IS_ | n | H_obs_ | F_IS_ |
|  |  |  |  |  |  |  |  |  |  |  |  |  |
| 301CT1 | 9 | 0.321 | 0.613* | 4 | 0.083 | 0.893* | 8 | 0.185 | 0.774* | 3 | 0.000 | 1.000* |
| 1132CT | 16 | 0.278 | 0.677* | 11 | 0.786 | 0.077 | 17 | 0.823 | -0.024 | 9 | 0.929 | -0.123 |
| 462GA1 | 14 | 0.552 | 0.373* | 6 | 0.333 | 0.605 | 11 | 0.526 | 0.357* | 6 | 0.333 | 0.600 |
| 109CT1 | 13 | 0.257 | 0.552* | 8 | 0.545 | 0.4 | 12 | 0.727 | 0.142 | 3 | 0.076 | 0.733 |
| 328CTT | 6 | 0.325 | 0.539* | 2 | 0.25 | 0.391 | 5 | 0.333 | 0.561* | 3 | 0.000 | 1.000 |
| 470AG1 | 8 | 0.382 | 0.402* | 4 | 0.6 | 0.11 | 4 | 0.342 | 0.422 | 3 | 0.692 | -0.180 |
| 71CGT1 | 3 | 0.167 | 0.059 | 1 | NA | NA | 2 | 0.342 | -0.067 | 2 | 0.231 | -0.091 |
| 766ATT | 5 | 0.447 | -0.067 | 3 | 0.6 | -0.156 | 4 | 0.432 | 0.129 | 3 | 0.500 | -0.189 |
| 176TG1 | 14 | 0.701 | 0.234* | 9 | 0.688 | 0.177 | 13 | 0.694 | 0.231* | 7 | 0.786 | 0.000 |
| 12ACG1 | 5 | 0.416 | 0.011 | 4 | 0.647 | -0.067 | 4 | 0.634 | -0.174 | 5 | 0.429 | 0.232 |
| 301ACG | 5 | 0.306 | 0.458* | 2 | 0.067 | 0.851 | 4 | 0.105 | 0.81* | 5 | 0.429 | 0.368 |
| 86AC1 | 3 | 0.26 | 0.3 | 2 | 0.063 | 0.776 | 3 | 0.105 | 0.662* | 2 | 0.538 | -0.333 |
| 88AT1 | 11 | 0.857 | -0.031 | 9 | 0.813 | 0.085 | 10 | 0.552 | 0.307* | 9 | 1.000 | -0.114 |
| 201AAT | 7 | 0.675 | 0.073 | 5 | 0.375 | 0.528 | 6 | 0.718 | -0.002 | 6 | 0.846 | -0.086 |
| 470CT2 | 6 | 0.221 | 0.101 | 4 | 0.214 | -0.04 | 3 | 0.139 | -0.048 | 3 | 0.357 | -0.140 |
| 71AT1 | 2 | 0.125 | 0.569* | 2 | 0.143 | -0.04 | 2 | 0.231 | 0.060 | 3 | 0.231 | -0.059 |
| 12ATG1 | 7 | 0.653 | -0.022 | 6 | 0.688 | 0.073 | 5 | 0.763 | -0.067 | 4 | 0.692 | 0.000 |
| 145TAA | 8 | 0.128 | 0.802* | 7 | 0.5 | 0.404 | 9 | 0.316 | 0.543* | 6 | 0.000 | 1.000* |
| 288CTA | 3 | 0.487 | -0.286 | 3 | 0.133 | -0.018 | 3 | 0.306 | 0.084 | 2 | 0.222 | 0.407 |
| 69TGA1 | 4 | 0.282 | -0.067 | 4 | 0.533 | 0.034 | 4 | 0.297 | -0.019 | 3 | 0.272 | 0.178 |
|  |  |  |  |  |  |  |  |  |  |  |  |  |
| Mean across loci | 7.45 | 0.392 | 0.311 | 4.8 | 0.424 | 0.296 | 6.45 | 0.4285 | 0.267 | 4.35 | 0.428 | 0.237 |
|  |  |  |  |  |  |  |  |  |  |  |  |  |
|  | **Ca-Ira** |  |  | **Barriere-Jeudy** | |  | **Chawa** |  |  | **Combined Popluations** | | |
|  |  |  |  |  |  |  |  |  |  |  |  |  |
| Locus | n | H_obs_ | F_IS_ | n | H_obs_ | F_IS_ | n | H_obs_ | F_IS_ | n | H_obs_ | PIC^d^ |
|  |  |  |  |  |  |  |  |  |  |  |  |  |
| 301CT1 | 5 | 0.167 | 0.761* | 4 | 0.2 | 0.689* | 7 | 0.172 | 0.785* | 11 | 0.205 | 0.783 |
| 1132CT | 14 | 0.917 | -0.015 | 8 | 0.742 | -0.014 | 9 | 0.021 | 0.976* | 24 | 0.536 | 0.866 |
| 462GA1 | 8 | 0.4 | 0.499* | 8 | 0.433 | 0.469* | 10 | 0.5 | 0.375* | 14 | 0.479 | 0.832 |
| 109CT1 | 3 | 0.381 | -0.152 | 3 | 0.154 | 0.286 | 3 | 0.314 | 0.120 | 14 | 0.348 | 0.586 |
| 328CTT | 3 | 0 | 1* | 4 | 0 | 1* | 5 | 0.212 | 0.706* | 7 | 0.200 | 0.593 |
| 470AG1 | 3 | 0.517 | 0.069 | 4 | 0.633 | -0.101 | 4 | 0.475 | 0.332* | 8 | 0.471 | 0.557 |
| 71CGT1 | 3 | 0.326 | -0.17 | 3 | 0.419 | -0.226 | 5 | 0.17 | -0.054 | 5 | 0.243 | 0.207 |
| 766ATT | 3 | 0.5 | 0.064 | 4 | 0.258 | 0.118 | 3 | 0.298 | 0.177 | 5 | 0.417 | 0.401 |
| 176TG1 | 10 | 0.915 | -0.177 | 9 | 0.429 | 0.352 | 13 | 0.844 | -0.018 | 15 | 0.738 | 0.867 |
| 12ACG1 | 5 | 0.63 | -0.284 | 4 | 0.2 | 0.087 | 5 | 0.255 | -0.089 | 6 | 0.449 | 0.408 |
| 301ACG | 5 | 0.438 | 0.162 | 5 | 0.452 | 0.247 | 5 | 0.413 | 0.258 | 5 | 0.330 | 0.539 |
| 86AC1 | 3 | 0.583 | -0.179 | 2 | 0.806 | -0.667* | 4 | 0.667 | -0.308 | 5 | 0.429 | 0.346 |
| 88AT1 | 8 | 0.77 | 0.007 | 9 | 0.903 | -0.047 | 12 | 0.933 | -0.062 | 14 | 0.821 | 0.844 |
| 201AAT | 4 | 0.729 | 0.012 | 6 | 0.742 | -0.021 | 5 | 0.733 | -0.026 | 8 | 0.699 | 0.694 |
| 470CT2 | 3 | 0.064 | 0.384 | 4 | 0.097 | 0.231 | 3 | 0.09 | 0.395 | 6 | 0.150 | 0.171 |
| 71AT1 | 2 | 0.291 | 0.421 | 2 | 0.267 | 0.27 | 3 | 0.295 | 0.383 | 3 | 0.223 | 0.311 |
| 12ATG1 | 6 | 0.787 | -0.057 | 6 | 0.484 | 0.191 | 6 | 0.711 | -0.128 | 7 | 0.687 | 0.675 |
| 145TAA | 4 | 0 | 1* | 4 | 0 | 1 | 4 | 0 | 1* | 11 | 0.113 | 0.637 |
| 288CTA | 2 | 0.667 | -0.492 | 3 | 0.677 | -0.324 | 3 | 0.682 | -0.320 | 3 | 0.521 | 0.358 |
| 69TGA1 | 4 | 0.167 | 0.054 | 3 | 0.129 | -0.034 | 3 | 0.159 | 0.067 | 4 | 0.239 | 0.232 |
|  |  |  |  |  |  |  |  |  |  |  |  |  |
| Mean across loci | 4.9 | 0.462 | 0.135 | 4.75 | 0.401 | 0.198 | 5.6 | 0.397 | 0.286 | 8.8 | 0.415 | 0.545 |

^a^n: number of alleles; ^b^H_obs_: observed heterozygosity; ^c^F_IS_: inbreeding coefficient; ^d^PIC: allelic polymorphic information content.

*denotes significant deviation from Hardy-Weinberg expectations based on Bonferroni adjusted significance levels; positive value indicates heterozygote deficit; negative value indicates heterozygote excess.
