# Supplementary material for: Genome-based polymorphic microsatellite development and validation in the mosquito Aedes aegypti and application to population genetics in Haiti
Source: BMC Genomics. 2009 Dec 9;10:590. doi: 10.1186/1471-2164-10-590 (PMC3087561; doi:10.1186/1471-2164-10-590)
Supplement: Additional file 4 — Regression analysis of pairwise FST/(1-FST) against pairwise natural logarithm-transformed distances among sample sites in Haiti. The data provided represent the comparison of microsatellite-based population structure to distances between populations in Haiti. [file 1471-2164-10-590-S4.DOC]

**Additional File 4. Regression analysis of pairwise F_ST_/(1-F_ST_) against pairwise natural logarithm-transformed distances among sample sites in Haiti.**
