# Supplementary material for: Genome-based polymorphic microsatellite development and validation in the mosquito Aedes aegypti and application to population genetics in Haiti
Source: BMC Genomics. 2009 Dec 9;10:590. doi: 10.1186/1471-2164-10-590 (PMC3087561; doi:10.1186/1471-2164-10-590)
Supplement: Additional file 5 — GenBank accession numbers for STS sequences of microsatellite loci. The data provided represent the complete list of single copy microsatellite loci with the associated GenBank Sequence Tagged Site (STS) database accession numbers. [file 1471-2164-10-590-S5.DOC]

**Additional File 5. GenBank accession numbers for STS sequences of microsatellite loci.**

| **Microsatellite locus** | **GenBank accession #** |
| --- | --- |
| 176CAT1 | GF101909 |
| 176TG1 | GF101910 |
| 12ACG1 | GF101911 |
| 12ATG1 | GF101912 |
| 71CGT1 | GF101913 |
| 71AT1 | GF101914 |
| 344ATTT1 | GF101915 |
| 192TAAA1 | GF101916 |
| 335CGA1 | GF101917 |
| 12CGT1 | GF101950 |
| 88CA1 | GF101918 |
| 88GAA1 | GF101919 |
| 673TA1 | GF101920 |
| 68ATGG1 | GF101921 |
| 68GAC1 | GF101922 |
| 440AAC1 | GF101923 |
| 440ATCC1 | GF101924 |
| 440TGTA1 | GF101925 |
| 145TAAA1 | GF101926 |
| 25AAG1 | GF101927 |
| 121GA1 | GF101928 |
| 328CTT1 | GF101929 |
| 1132CT1 | GF101930 |
| 301ACG1 | GF101931 |
| 301CT1 | GF101932 |
| 17ATA1 | GF101933 |
| 766ATT1 | GF101934 |
| 69TGA1 | GF101935 |
| 217CTT1 | GF101951 |
| 201TTA1 | GF101936 |
| 201AAT1 | GF101937 |
| 470CT2 | GF101938 |
| 470AG1 | GF101939 |
| 12CAA1 | GF101940 |
| 446CCA1 | GF101941 |
| 68ATT1 | GF101942 |
| 438A1 | GF101943 |
| 319A1 | GF101944 |
| 25TTAT1 | GF101945 |
| 29AT1 | GF101958 |
| 14GCT1 | GF101946 |
| 288CTA1 | GF101947 |
| 69AT1 | GF101948 |
| 69CA1 | GF101949 |
